# Development of a replication competent murine norovirus reporter system

**DOI:** 10.1371/journal.ppat.1012834

**Published:** 2025-05-22

**Authors:** Mikayla C. Olson, Linley R. Pierce, Robert C. Orchard

**Affiliations:** Department of Immunology, University of Texas Southwestern Medical Center, Dallas, Texas, United States of America; US Food and Drug Administration, UNITED STATES OF AMERICA

## Abstract

Caliciviruses are significant agricultural and human pathogens that are poorly understood due to the dearth of molecular tools, including reporter systems. We report the development of a robust luciferase-based reporter system for a model calicivirus, murine norovirus (MNoV). Genetic insertion of a HiBiT tag, an 11 amino acid fragment of nanolucifersase, at the junction of the nonstructural proteins NS4 and NS5 yields infectious virus. The resultant MNoV-HiBiT produces a robust signal that is detected early in infection and occurs only in cells susceptible to MNoV infection. The MNoV-HiBiT reporter is effective at monitoring acute infection in STAT1 deficient mice. Furthermore, we used this tool to characterize two unappreciated host directed anti-MNoV compounds. The use of the MNoV-HiBiT virus enables new mechanistic studies by a rapid and quantitative means of measuring MNoV replication. The HiBiT insertion strategy we describe may be useful for the generation of other calicivirus reporters.

## Author summary

Colloquially known as “the stomach bug,” human norovirus is a leading cause of foodborne illness worldwide. Despite its prevalence, there are no approved therapeutics or vaccines against norovirus owing to the challenges in cultivation of the virus and a lack of a small animal model of human norovirus. Murine Norovirus (MNoV) has emerged as a tractable model due to its similarity to human norovirus and the ability to grow the virus in laboratory strains of mice and immortalized cells in vitro. Reporter viruses, which include enzymatic or fluorescent reporter genes inserted in the viral genome, are invaluable tools that expedite viral research. Unfortunately, there are no MNoV reporter systems available. Here we developed a novel reporter MNoV using a complementary nanoluciferase. Specifically, we inserted a HiBiT tag into the MNoV genome that is functionalized into a full nanoluciferase protein and emits light in the presence of LgBiT. We demonstrated that MNoV-HiBiT is a faithful and sensitive reporter of viral replication across MNoV strains and cell types as well as in vivo. We used the reporter virus to screen novel anti-viral compounds, demonstrating that the MNoV-HiBiT system is a powerful tool to enhance norovirus research.

## Introduction

Human Norovirus (HNoV), a positive-sense RNA virus of the *Calicivirdae* family, is the leading cause of gastroenteritis worldwide [1]. Currently, there is no approved vaccine or therapeutic for HNoV, largely due to our limited understanding of calicivirus biology [1]. For many viral systems, the development of reporter viruses has greatly enhanced our understanding of the viral life cycle and accelerated the development of therapeutics and vaccines. Reporter systems for caliciviruses are lacking. Unlike HNoV, murine norovirus (MNoV) replicates in immortalized tissue culture cell lines and can infect laboratory strains of mice, making it an excellent model system for caliciviruses [2,3]. Despite a robust reverse genetic system, the incorporation of fluorescent or enzymatic reporters into the MNoV genome has not been successful. Recently, a single cycle MNoV reporter system was developed through a trans complementation approach [4]. However, a replication competent reporter system for caliciviruses has yet to be achieved and would advance research progress. We hypothesized MNoV might tolerate an insertion of a small reporter at the junction between nonstructural proteins 4 and 5 (NS4-5), as this site is poorly processed by noroviruses [5]. Here, we describe such a system using a complementary luciferase approach that accurately reports on MNoV replication.

## Results

**Fig 1:**
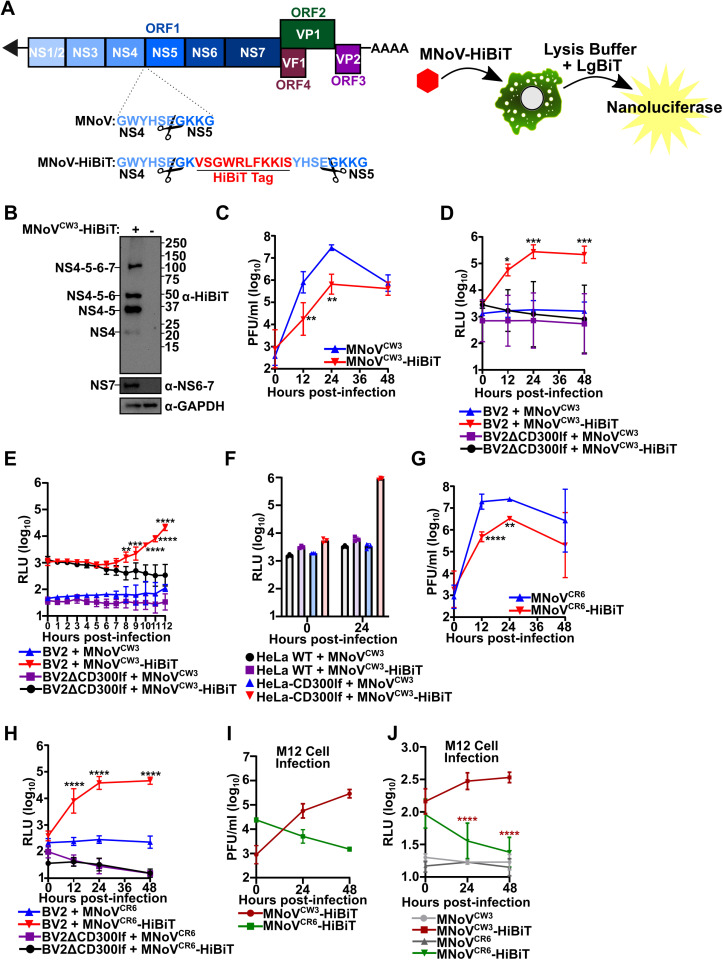
Development and Characterization of MNoV-HiBiT Viruses. **A)** (Left) Cartoon illustration of the MNoV genome organization with a focus on the NS4-NS5 cleavage site and the introduction of the HiBiT tag (red). Scissors indicate the MNoV protease cleavage site. (Right) Cartoon illustration of the utility of the MNoV-HiBiT virus and the complementation with LgBiT supplied in lysis buffer to form a functional nanoluciferase. **B)** Representative western blot of BV2 cells either uninfected or infected with MNoV^CW3^-HiBiT at a multiplicity of infection (MOI) of 1.0 for 12 hours and probed for indicated antibodies. Predicted molecular weight of MNoV nonstructural proteins are indicated. **C)** BV2 cells were challenged with MNoV^CW3^ or MNoV^CW3^-HiBiT at an MOI of 0.05. Viral production was enumerated using plaque assays (PFU; plaque forming units) at the indicated time points. D-E) BV2 or BV2ΔCD300lf cells were challenged with MNoV^CW3^ or MNoV^CW3^-HiBiT at a multiplicity of infection (MOI) of 5.0. At the indicated time points, infection was quantified using Nano-Glo HiBiT Lytic Detection System (RLU; relative luminescent units). **F)** HeLa or HeLa-CD300lf cells were challenged with MNoV^CW3^ or MNoV^CW3^-HiBiT at an MOI of 1.0 and at the indicated time points, infection was quantified using Nano-Glo HiBiT Lytic Detection System. **G)** BV2 cells were challenged with MNoV^CR6^ or MNoV^CR6^-HiBiT at an MOI of 0.05. Viral production was enumerated using plaque assays at the indicated time points. **H)** BV2 or BV2ΔCD300lf cells were challenged with MNoV^CR6^ or MNoV^CR6^-HiBiT at a multiplicity of infection (MOI) of 0.5. At the indicated time points, infection was quantified using Nano-Glo HiBiT Lytic Detection System. **I)** M12 cells were challenged with MNoV^CW3^-HiBiT or MNoV^CR6^-HiBiT at an MOI of 0.05. Viral production was enumerated using plaque assays (PFU; plaque forming units) at the indicated time points. Data from two independent experiments **J)** M12 cells were challenged with indicated viral strains at an MOI of 0.1 At the indicated time points, infection was quantified using Nano-Glo HiBiT Lytic Detection System. Unless otherwise noted, all data are shown as mean ± S.D. from three independent experiments and analyzed by one way ANOVA with Tukey’s multiple comparison test. Statistical significance is annotated as follows: ns not significant, * P < 0.05, ** P < 0.01, *** P < 0.001, **** P < 0.0001.

We were inspired by the previously generated T-cell epitope viruses where the small peptide SIINFEKL or the GP33 epitope is inserted between MNoV nonstructural proteins 4 and 5 (NS4 and NS5) with a duplicated protease cleavage site [6,7]. To generate a replication competent reporter system, we inserted the small HiBiT peptide sequence at the identical site into the genome of MNoV^CW3^ (herein called MNoV^CW3^-HiBiT; Fig 1A). The eleven amino acid HiBiT tag can be functionalized into nanoluciferase by the addition of LgBiT in cells or in the luciferase lysis buffer [8]. The latter enables the use of HiBiT system in any cell type without the need to modify the cells. The HiBiT epitope is detected on NS4–5 polyprotein precursors and to a small extent on processed NS4. (Fig 1B). The molecular clone of MNoV^CW3^-HiBiT produced infectious virus. Insertion of the HiBiT sequence significantly diminished MNoV^CW3^-HiBiT replication but still enabled exponential growth of the virus compared to parental MNoV^CW3^ (Fig 1C). To assess the functionality and specificity of MNoV^CW3^-HiBiT in vitro, we performed a time course luciferase assay using either wildtype BV2 cells or BV2ΔCD300lf cells, which lack the receptor necessary for MNoV infection [9]. Cells were inoculated with either the parental MNoV^CW3^ or MNoV^CW3^-HiBiT virus and lysed after a single cycle of infection (12 hours) or multiple cycles of infection (24 or 48 hours). We then measured the luminescence from the cell lysate by supplying the LgBiT via the lysis buffer *in trans*. Infection of wild-type BV2 cells with MNoV^CW3^-HiBiT led to exponential increase in light production by luciferase with nearly a 1000-fold difference in RLU at 48 hours post-infection (Fig 1D). This signal was specific to the HiBiT virus and required entry of MNoV (Fig 1D). To further assess the sensitivity of the reporter virus, we conducted another time course assay with timepoints at 1-hour increments for 12 hours. Statistical differences in luminescence for MNoV^CW3^-HiBiT in wildtype BV2 cells compared to controls were detected as early as at 8 hours post-infection (Fig 1E). Additionally, we validated that MNoV^CW3^-HiBiT is infectious and luminescent in human cells when CD300lf is ectopically expressed. Wildtype HeLa cells, which lack the MNoV receptor, or HeLa-CD300lf cells were inoculated with MNoV^CW3^ or MNoV^CW3^-HiBiT. The HeLa-CD300lf cells infected with MNoV^CW3^-HiBiT produced 1000-fold more RLUs than controls (Fig 1F). Taken together, these data demonstrate that the MNoV^CW3^-HiBiT virus is specific in monitoring viral replication by luminescent output.

MNoV strains exhibit different infection outcomes after oral inoculation [10]. For example, MNoV^CW3^ causes an acute infection that spreads to extraintestinal tissues and is lethal in STAT1 deficient animals. In contrast, MNoV^CR6^ establishes a persistent infection in the gastrointestinal tract and does not spread or cause lethality in immunodeficient animals [10,11]. We set out to determine if our reporter system works across MNoV viral strains and target cells *in vitro*. First, we generated an MNoV^CR6^-HiBiT virus analogous to the one generated with MNoV^CW3^ described above. MNoV^CR6^-HiBiT is also attenuated compared to the parental virus (Fig 1G). MNoV^CR6^-HiBiT had exponential growth of RLUs both at single and multicycle timepoints, indicating that this tagging strategy can be used for multiple MNoV strains (Fig 1H). The amount of MNoV infection of B cells varies by strain due to genetic variation in capsid [12]. Consistent with these reports, we observe differential infection outcomes of MNoV^CW3^-HiBiT and MNoV^CR6^-HiBiT infection of the murine B-cell line M12 as measured by plaque-forming units and luminescence (Fig 1I and 1J). Specifically, we detect modest infection by MNoV^CW3^-HiBiT but relatively no infection by MNoV^CR6^-HiBiT in M12 cells (Fig 1J). Thus, these data indicate that the HiBiT reporter system can be useful to study MNoV strain differences in vitro.

**Fig 2:**
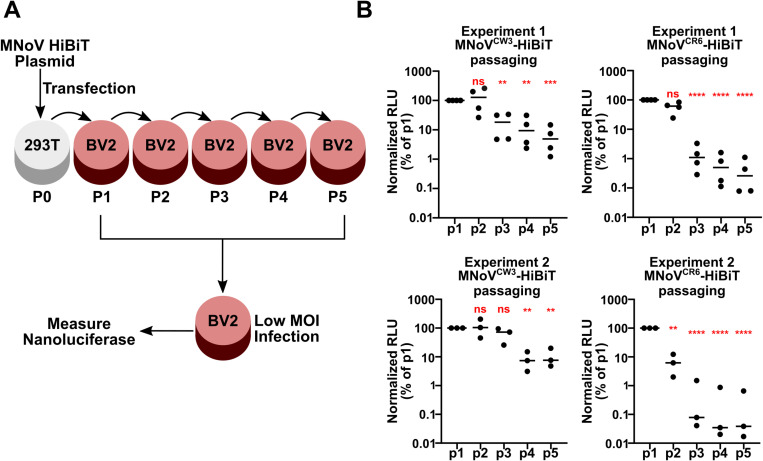
Stability of the HiBiT reporter during repeated passaging. **A)** Cartoon schematic of the passaging strategy. First, plasmids containing the MNoV-HiBiT molecular clone are transfected into HEK293T cells and subsequently passaged onto BV2 cells. Passaged stocks are titered and tested for luminescence capacity via low MOI infection of BV2 cells. **B)** Two independent passaging experiments with MNoV^CW3^-HiBiT (Left) and MNoV^CR6^-HiBiT (Right) with luminescence values being normalized to the initial p1 stock. RLU quantified using Nano-Glo HiBiT Lytic Detection System. All data are shown as mean ± S.D. from three or four independent experiments and analyzed by one way ANOVA with Tukey’s multiple comparison test. Statistical significance is annotated as follows: ns not significant, * P < 0.05, ** P < 0.01, *** P < 0.001, **** P < 0.0001.

Because the HiBiT containing MNoV viruses are attenuated in viral replication (Fig 1C and 1G), we wanted to determine how stable the HiBiT insertion is after multiple passages. We devised a passaging scheme in which MNoV^CW3^-HiBiT or MNoV^CR6^-HiBiT are serially passaged through BV2 cells at low multiplicities of infection (MOI), mimicking the generation of viral stocks. Virus was collected at each passage, titered, and assessed for nanoluciferase activity after a subsequent 24-hour infection of BV2 cells at a low MOI (Fig 2A). We conducted two independent passaging experiments for both MNoV^CW3^-HiBiT and MNoV^CR6^-HiBiT. Each passaged stock was assessed three to four independent times for luminescence. In all cases luminescence outputs dropped precipitously by the fourth passage (Fig 2B). In most cases the decrease in luminescence was apparent in the second or third passage (Fig 2B). These data are consistent with a strong selective pressure for replication due to attenuation of the inserted HiBiT sequence and cautions against serial passaging of MNoV-HiBiT viruses (Fig 1C and 1G).

**Fig 3:**
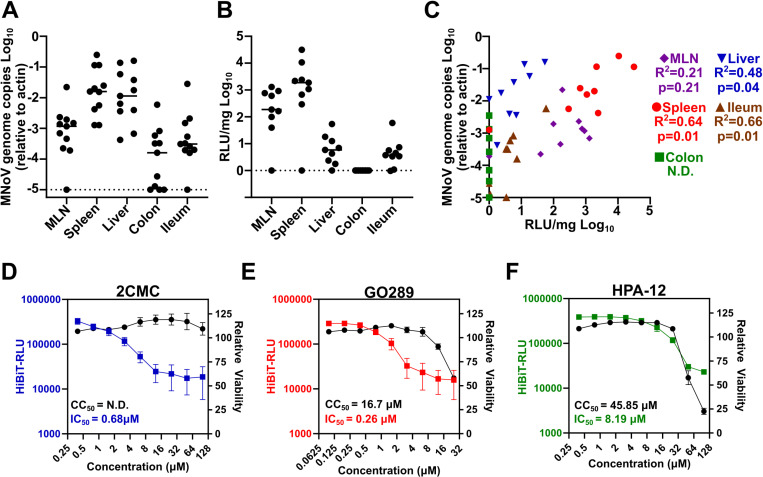
Utility of the MNoV-HiBiT system for in vivo applications and antiviral discovery. A-B) STAT1^-/-^ mice were inoculated with 2.5 x 10^5^ PFU of MNoV^CW3^-HiBiT and euthanized 3 days post-infection. **(A)**Tissue titers for mesenteric lymph nodes (MLN), spleen, liver, colon, and ileum were analyzed by qPCR for MNoV genome copies and normalized to actin. **(B)** Relative Luminescence Units (RLU) from indicated tissue homogenates quantified using Nano-Glo HiBiT Lytic Detection System. Raw RLU values from uninfected matched tissues were used to background subtract from infected samples. Dotted lines represent limit of detection of both assays. **C)** Correlation of MNoV genome copies (y-axis) and RLU (x-axis) for indicated tissues. R^2^ and P values for linear regression analyses for each tissue are included in the legend. D-F) Dose response curves of BV2 cells treated with indicated doses of 2CMC **(D)** GO289 **(E)**, or HPA-12 (F) infected with MNoV^CW3^-HiBiT (left axis) or uninfected and measured for cell viability using CellTiterGlo (right axis). Inhibitory Concentration 50 (IC_50_) and Cytotoxic Concentration 50 (CC_50_) are listed in bottom left corner. All data are shown as mean ± S.D. from three independent experiments.

An important facet of the MNoV model system is the capacity to infect laboratory strains of mice. However, there is not a transgenic mouse that expresses LgBiT for in vivo luciferase complementation of MNoV-HiBiT viruses currently available. Therefore, to test the utility of the MNoV-HiBiT reporter in vivo, we infected STAT1 deficient animals with MNoV^CW3^-HiBiT. Three days post-infection we harvested tissues to detect infection by quantifying MNoV genomes or measuring luciferase activity from tissue homogenates supplied with LgBiT *in trans*. Genomic copies and luminescence values were robustly detected in the mesenteric lymph nodes (MLNs), spleens, livers, and ilea of MNoV^CW3^-HiBiT infected animals (Fig 3A and 3B). While genomic copies were detected in the colon, only background levels of luminescence were detected (Fig 3A and 3B). Overall, the correlation between luminescence and genomic copies was statistically significant across multiple tissues (Fig 3C). The notable exception is the colon. Whether this represents a sensitivity issue with the HiBiT system, the lysis conditions of the colon, or with the stability of the HiBiT insertion remains unclear. Even so, our data indicates that the MNoV^CW3^-HiBiT reporter system can be translated for in vivo use.

Reporter viruses enable the assessment and screening of genetic or chemical perturbations at a scale that is challenging with traditional viral titer measurements. To demonstrate the utility of MNoV-HiBiT to identify new chemical inhibitors of norovirus replication, we mined a previously conducted CRISPR/Cas9 loss of function screen for MNoV dependency factors that have small molecule inhibitors [13]. We focused on Casein Kinase II (CKII) and CERT1 (also known as COL4A3BP) which have well characterized inhibitors: GO289 and HPA-12 respectively. Importantly, these molecules and their respective targets have not been validated to have anti-norovirus activity. We compared the antiviral activity of these compounds to the nucleoside analog 2′-C-methylcytidine (2CMC), which has known anti-norovirus activity [14, 15]. 2CMC inhibited MNoV^CW3^-HiBiT infection of BV2 cells with an IC_50_ of 0.68 µM, a value similar to that described previously for MNoV (Fig 3D) [14]. Treatment of BV2 cells with the CKII inhibitor GO289 strongly inhibited MNoV replication with minimal toxicity (Fig 3E). Treatment of cells with the CERT inhibitor HPA-12 demonstrated a more modest reduction in viral replication ~5.5 fold more potent than its cytotoxic effects (Fig 3F). Overall, these data demonstrate the utility of the MNoV-HiBiT system for identification and characterization of small molecule inhibitors of MNoV.

## Discussion

Norovirus imposes a global health burden every year [16]. As such, novel tools to study viral replication and discover new therapeutics are necessary. Here, we have developed a replication competent reporter MNoV. MNoV-HiBiT confers a broad range of utility as the LgBiT can be added in the lysis buffer, eliminating the need for specialty cell lines. However, there are a few limitations of our current reporter system. First, the MNoV-HiBiT virus is significantly attenuated and thus caution is necessary in propagating these viruses to avoid losing the inserted sequence (Fig 2). Nonetheless, MNoV-HiBiT still has robust infection as evidenced by multiple MNoV strains and different cellular systems that we successfully interrogated. To this point, infection of STAT1^-/-^ mice with MNoV^CW3^-HiBiT revealed a high level of correlation between genome copies and luminescence values, with one notable exception, the colon (Fig 3C). Interestingly, this data parallel studies in BALB/C mice where replicated virus was absent in the colon but input virions were detected [17]. Thus, it is possible that HiBiT luminescence is faithfully reporting a lack of viral replication in the colon. While we demonstrated that the luminescence output from MNoV^CW3^-HiBiT can be used to probe replication kinetics after infection of STAT1 deficient mice, we were unable to assess in vivo bioluminescent imaging given the lack of a LgBiT expressing mouse line. The current reporter systems will also need to be optimized in the context of other MNoV infection models such as neonatal diarrhea or persistent infection of adult immunocompetent mice [10, 18]. Additionally, the dual cleavage sites present uncertainty of the extent to which NS4 and NS5 are labeled compared to unlabeled, as we do not have specific antibodies targeting these nonstructural proteins. We did not explore whether other insertion sites or strategies in the MNoV genome would have equal or superior sensitivity to the current design. The NS4-NS5 cleavage site is poorly processed across norovirus genogroups, including human norovirus [5]. Thus, it is possible that this insertion strategy may be broadly applicable to related caliciviruses. Our data demonstrates that the MNoV-HiBiT reporter system can be useful across a multitude of applications. Elucidation of viral mechanisms, large scale screens, and antiviral drug validation, as shown here, are among the many opportunities for which MNoV-HiBiT can be instrumental.

## Materials and methods

### Ethics statement

Animal work described in this manuscript has been approved and conducted under the oversight of the University of Texas Southwestern Institutional Animal Care and Use Committee under protocol 2018-102627.

### Cell culture

293T (ATCC), Hela (ATCC), M12 cells (Kind gift of Dr. Skip Virgin, Washington University) and BV2 cells (Kind gift of Dr. Skip Virgin, Washington University) were cultured in Dulbecco’s Modified Eagle Medium (DMEM) with 5% fetal bovine serum (FBS). BV2∆CD300lf and HeLa-CD300lf cells have been described previously [9]. 10 µg/mL of blasticidin (Thermo Fisher) was added for the maintenance of HeLa-CD300lf cells. Cell lines were tested regularly and verified to be free of mycoplasma contamination.

### Construction of MNoV-HiBiT

Plasmids encoding MNoV-HiBiT were generated in MNoV^CW3^ (Gen bank accession no. EF014462.1) or MNoV^CR6^ (Gen bank accession no. JQ237823) appropriately. The HiBiT tag with a cleavage site sequence on both sides was inserted between the coding regions for NS4 and NS5 (S1 and S2 Figs). DNA constructs were all verified by sequencing. MNoV-HiBiT plasmids were transfected into 293Ts (p0) and amplified on BV2s (p1) until at least 50% cytopathic effect (CPE) was observed, as described previously [9].

### MNoV growth assay

MNoV growth curve assays were conducted by seeding 5 x 10^4^ BV2 or M12 cells in 96-well plates. Infection was initiated by the concurrent addition of virus (MOI 0.05) with cell plating. Plates were frozen at -80C at the appropriate timepoints before performing plaque assay as previously described [9]. Briefly, 2.5 x 10^5^ BV2 cells were seeded per well of 24-well plate. Media was removed the following day, and 10-fold serial dilutions of lysate were added to each well, incubated with gentle rocking for 1 hour, and then removed. Cells were overlayed with complete media containing 1% methylcellulose. Plates were incubated for 3 days before staining with crystal violet solution.

### *in vitro* Luciferase assay

5 x 10^4^ BV2 or 2 x 10^4^ HeLa cells were seeded in 96-well plates and the next day inoculated with virus (MOI 5.0 for MNoV^CW3^-HiBiT and MOI 0.5 for MNoV^CR6^-HiBiT). For M12 cells, 5 x 10^4^ M12 cells were seeded and infected concurrently at an MOI of 0.1. Plates were frozen at the indicated time points at -80°C. After thawing, luminescent activity was measured with a Synergy LX multi-mode reader (Biotek) using a Nano-Glo HiBiT Lytic Detection System (Promega N3040) following the manufacturer’s protocols. Briefly, 100 µL of Nano-Glo HiBiT Lytic Buffer containing LgBiT and luciferase substrate are added to the wells and incubated at room temperature for 10 minutes prior to measuring luminescence.

### Western blotting

Cells were lysed in 2x Lamelli buffer (BioRad Cat. #1610737) with 5% β-Mercaptoethanol. Lysates were boiled and centrifuged prior to resolution on SDS-PAGE gels and transfer to a PVDF membrane. Membranes were probed with α-HiBiT (Promega 1:1000), α-NS6/7 (1:5,000), and α-GAPDH (Sigma, 1:1000).

### Passaging

3 x 10^6^ BV2 cells were plated in a T25 flask and immediately infected with p1 stocks of MNoV^CW3^-HiBiT or MNoV^CR6^-HiBiT at an MOI of 0.05. 30–48 hours post-infection flasks were frozen at -80°C. After thawing, samples were clarified via centrifugation at 4,000 *g* for 10 minutes and subsequently aliquoted. 100 µL of this p2 virus was then used to inoculate a fresh T25 flask seeded with 3 x10^6^ BV2 cells. This process was repeated until p5 was reached. Two independent passaging experiments with each strain of the virus were conducted. PFU per ml for each passaged virus was determined via plaque assay. For determining luminescence capacity of the viral strains, 5 x 10^4^ BV2 cells were seeded and immediately infected with different passaged viruses at a fixed MOI of 0.05. 24 hours post-infection, plates were frozen and then assayed for luminescence as described above.

### Mouse infections

2.5 x 10^5^ PFU of MNoV^CW3^-HiBiT was orally administered in 25uL of DMEM to 6- to 8-week-old Stat1^–/–^ [B6.129S(Cg)-Stat1tm1Div/J] mice. All mice were singularly housed after inoculation. Mice were euthanized at 3 days post infection and tissues were harvested.

RNA was extracted from tissues using TRI Reagent (Sigma- Aldrich # T9424) and a Direct-zol kit (Zymo Research R2052) according to the manufacturer’s protocols. cDNA was synthesized from 1 µg of RNA using the High-Capacity cDNA Reverse Transcription kit (Thermo Fisher Scientific # 4368813) according to the manufacturer’s protocols. MNoV TaqMan quantitative PCR was performed for each sample in triplicate using the forward primer 5’-GTGCGCAACACAGAGAAACG-3’, reverse primer 5’-CGGGCTGAGCTTCCTGC-3’, and probe 59-6FAM-CTAGTGTCTCCTTTGGAGCACCTA-BHQ1-3’. Actin TaqMan quantitative PCR was performed for each sample in triplicate using the forward primer 5’-GATTACTGCTCTGGCTCCTAG-3’, reverse primer 5’-GACTCATCGTACTCCTGCTTG-3’, and probe 5’-6FAM-CTGGCCTCACTGTCCACCTTCC-6TAMSp-3’.

Tissue fragments (up to 500 mg), including cleared colons, were homogenized in 200µL of RIPA buffer with Halt Protease and Phosphatase Inhibitor Cocktail (Thermo Scientific 78444). 50uL of tissue homogenate was mixed with 50uL Nano-Glo HiBiT Lytic Buffer containing luciferase substrate and LgBiT (Promega N3040). Tissue sample luciferase assays were performed in triplicate for each sample and values were background subtracted using matched uninfected tissue homogenates.

### Anti-viral screening

5 x 10^4^ BV2 cells per well were seeded in white-walled 96 well plates. At time of seeding, the cells were concurrently treated with indicated compound and either mock infected or infected with MNoV^CW3^-HiBiT at an MOI of 0.05. 24 hours post-infection, cells were subjected to HiBiT luciferase assay as described above. Viability of mock infected cells was determined using CellTiter-Glo (Promega) according to manufacturer’s instructions at 24 hours post infection.

## Supporting information

S1 Table Primary data for Figs. (XLSX)

S1 Fig Genbank file of the MNoV^CW3^-HiBiT plasmid. (TXT)

S2 Fig Genbank file of the MNoV^CR6^-HiBiT plasmid. (TXT)
